# Effects of autologous platelet-rich plasma on implantation and pregnancy in repeated implantation failure: A pilot study

**Published:** 2016-10

**Authors:** Leila Nazari, Saghar Salehpour, Sedighe Hoseini, Shahrzad Zadehmodarres, Ladan Ajori

**Affiliations:** *Department of Obstetrics and Gynecology, Preventive Gynecology Research Center, Shahid Beheshti University of Medical Sciences, Tehran, Iran.*

**Keywords:** *Platelet-rich plasma*, *Implantation*, *Fertilization in Vitro*, *Pregnancy rate*, *Repeated implantation failure*

## Abstract

**Background:**

Repeated implantation failure (RIF) is a major challenge in reproductive medicine and despite several methods that have been described for management, there is little consensus on the most effective one.

**Objective:**

This study was conducted to evaluate the effectiveness of platelet-rich plasma in improvement of pregnancy rate in RIF patients.

**Materials and Methods:**

Twenty women with a history of RIF who were candidates for frozen-thawed embryo transfer were recruited in this study. Intrauterine infusion of 0.5 ml of platelet-rich plasma that contained platelet 4-5 times more than peripheral blood sample was performed 48 hrs before blastocyst transfer.

**Results:**

Eighteen participants were pregnant with one early miscarriage and one molar pregnancy. Sixteen clinical pregnancies were recorded and their pregnancies are ongoing.

**Conclusion:**

According to this study, it seems that platelet-rich plasma is effective in improvement of pregnancy outcome in RIF patients.

## Introduction

Repeated implantation failure (RIF) is defined as failure to conceive following several embryo transfers in in vitro fertilization (IVF) cycles. There are no standard criteria for RIF description. According to European Society of Human Reproduction and Embryology consortium, RIF is defined as the absence of gestational sac on ultrasound at 5 wks or more after embryo transfer (ET) following 3 ET with high-quality embryos or after transfer of 10 or more embryos in multiple transfers (1-5). Numerous factors are involved in process of implantation including embryo quality, endometrial receptivity and immunological factors (6-8).

Several methods have performed for RIF management but there is little consensus on the most effective one. Blastocyst transfer, preimplantation genetic screening (PGS), assisted hatching, co-culture system, sequential transfer, hysteroscopy, endometrial scratching, salpingectomy for tubal disease, extra number embryo transfer, natural cycle, oocyte donation, intra-tubal ET, immune therapy and endometrial receptivity array (ERA) have been used but there is not any proved evidence in these treatments (9-12). 

Recently, intrauterine infusion of platelet-rich plasma (PRP) is described to promote endometrial growth and receptivity. PRP is prepared from fresh whole blood that contained several growth factors and cytokines including fibroblast growth factor (FGF), platelet derived growth factor (PDGF), vascular endothelial growth factor (VGEF), transforming growth factor (TGF), insulin-like growth factor I, II (IGF-I, II), connective tissue growth factor (CTGF) and interleukin 8 (IL-8). PRP has been investigated as a therapeutic approach for several medical disorders including nerve injury, ocular epithelial defects, alopecia, cardiac muscle injury, osteoarthritis, and tendinitis. Despite the wide use of PRP in several fields in medicine, it’s efficacy in obstetrics and gynecology is limited (13-18).

We designed the present study to investigate whether intrauterine infusion of PRP could improve pregnancy outcome in women with RIF. 

## Materials and methods

**Study design**

This was a single arm preliminary study of an RCT (registered at Iranian Registry of Clinical Trials: IRCT2016072229027N1) which conducted in IVF center, Taleghani Hospital, Tehran, Iran. The study was approved by ethical committee of Shahid Beheshti University of Medical Sciences (SBMU). All participants signed an informed written consent. 

**Figure 1:**
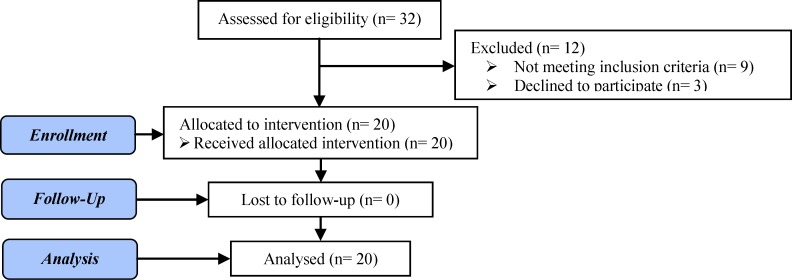
Flow Diagram

Thirty-two women who failed to conceive after 3 or more ET with high-quality embryos who were candidates for frozen-thawed embryo transfer (FET) were assessed for eligibility to enter the study from March to June 2016. Twelve women were excluded for different reasons; 20 were included in the study (Figure 1). The inclusion criteria were age below 40 yrs, body mass index (BMI) below 30 kg/m^2^. The exclusion criteria were hematological and immunological disorders, hormonal disorders, chromosomal and genetic abnormalities and uterine abnormalities (acquired or congenital). 

The hysteroscopic examination was performed before the cycle if it was not previously done. Laboratory evaluation of thrombophilia, antiphospholipid antibodies, hormonal disorders, hematological and immunological disorders in women and karyotype of couples were performed. All participants underwent FET cycle and hormone replacement therapy was performed for endometrial preparation as a same route: estradiol valerate (Aburaihan Co., Tehran, Iran) 6 mg/d was started from 2^nd^ or 3^rd^ day of mensural cycle and it was increased to 8 mg/d if endometrial thickness did not reach at least 8 mm.

During the cycle whenever the endometrial thickness was more than 8 mm, progesterone suppository (Cyclogest; Actavis, UK limited, England) 400 mg twice daily was started. Good quality blastocysts (Grade A or B according to embryologic scoring) transferred for all of the participants. Intrauterine infusion of PRP was done 48 hrs before ET. PRP was prepared from autologous blood and it was made by using two steps centrifuge process. Estradiol valerate and progesterone supplementation were continued for 2 wks after ET and if the serum Beta-Human Chorionic Gonadotropin (β-HCG) was positive hormone supplementations were continued until 12 wks of gestation. All Blastocyst transfers were performed under ultrasound guidance by one expert gynecologist with infertility fellowship. ET was performed according to American Society for Reproductive Medicine (ASRM) guidelines 2013 (Two or three embryos for each participant). On PRP infusion day, 17.5 ml of peripheral venous blood was drawn into a syringe that contains 2.5 ml of Acid Citrate A Anticoagulant solution (Arya Mabna Tashkhis, Iran) and centrifuged immediately at 1200 rpm for 12 min to separate red blood cells, then plasma was centrifuged again at 3300 rpm for 7 min to obtain PRP that contained platelet 4-5 times more than peripheral blood. 0.5 ml of PRP was infused into the uterine cavity with IUI catheter (Takwin, Iran). The previous cycle of each participant served as its own control.

**Outcome assessment**

Chemical pregnancy and clinical pregnancy was determined by positive serum β-HCG, 2 wks after ET and presence of fetal heart beat in transvaginal ultrasound 5 wks after ET.

## Results

A total of 20 participants with RIF history were entered into this study. All of them were able to complete the study and their data were analyzed. Table I provides baseline characteristics summary. Uterine cavity abnormalities were not detected before starting the cycles. Seven women had a history of hysteroscopic surgery due to septum, Asherman’s syndrome and submucosal myoma. Participants had a history of failed previous ET attempts between 3-7 and their mean age was 33.4±5.7 years. Eighteen participants were pregnant with one early miscarriage and one molar pregnancy. Sixteen clinical pregnancies were recorded and their pregnancies are ongoing. 

**Table I:** Patients’ characteristics

Age (years)*		33.4 ± 5.7
BMI (Kg/m^2^)*		26.3 ± 3.6
Etiology of Infertility**		
	Male Factor	12 (60)
	Female Factor	
	Anovulation	5 (19)
	DOR	7 (35)
	Tubal Factor	3 (15)
	Endometriosis	2 (10)
	Mixed (MF + FF) (%)	9 (45)
Previous failed ET Cycles (no) (Min-Max)		3-7

BMI: Body mass index

DOR: Diminished ovarian reserve

* Data presented as mean±SD.

** Data presented as n (%)

## Discussion

Despite expanding experience in advanced reproductive technologies and great improvement in infertility treatment, implantation failure is one of the major challenges (8, 20-22). The receptive endometrium is the main factor for implantation and pregnancy. Even in the cases of replacement of chromosomally normal embryos, confirmed by PGS, successful implantation and pregnancy are not reassuring (2). In a normal menstrual cycle in human, endometrium becomes receptive during the mid-secretory phase around days 19-23 that is described as window of implantation. During this period, cytokines, growth factors, prostaglandins, and adhesion molecules are expressed and inconsistency of these proteins could impair implantation and pregnancy. Sak and co-workers investigated that expression of growth factors in the endometrium of women with RIF history is less than normal fertile women (21, 23, 24). 

According to this hypothesis local infusion of PRP that contains several growth factors and cytokines may improve endometrial receptivity and implantation. PRP is collected from an autologous blood sample that has been enriched with platelets about 4-5 times more than circulating blood. PRP with a large amount of cytokines and growth factors can stimulate proliferation and regeneration. Recently, Chang reported the efficacy of PRP intrauterine infusion in endometrial growth in the refractory thin endometrium. Five participants in whom embryo transfer was canceled due to thin endometrium underwent intrauterine infusion of PRP. Adequate endometrial growth and pregnancy were reported in all of them and pregnancy was normally progressed in 4 women (13, 25-28).

Just recently, Reghini and co-workers suggested the efficacy of PRP for the treatment of inflammatory response in chronic degenerative endometritis in mares. In this trial, 13 mares with endometrium classified as chronic degenerative endometritis and 8 mares with normal endometrial histology were selected to investigate the PRP therapy effect. The mares were inseminated with fresh semen in two consecutive cycles in a crossover study design. Each mare served as its own control and the treatment was performed with intrauterine PRP infusion four hrs after artificial insemination. They concluded that PRP was effective in modulating the exacerbated uterine inflammatory response to semen in mares with chronic degenerative endometritis (29). 

The result of our study revealed the efficacy of PRP intrauterine infusion on implantation and pregnancy. After PRP, 18 RIF patients were pregnant with 16 ongoing pregnancies and only one miscarriage and one molar pregnancy. Currently, PRP is safely used in several conditions in medicine. It is achieved from autologous blood sample and risks of immunological reaction and transmission of infections are eliminated. As we know, this is the first study that evaluated the efficacy of PRP intrauterine infusion in RIF patients and based on the results, it is suggested to design further randomized clinical trials in this field. According to several factors that involved in implantation process, we excluded a large number of women with a history of RIF in our trial and it was a limiting factor.

## Conclusion

According to this study, it seems that PRP is effective in improvement of pregnancy outcome in RIF patients.
